# The missing hallmark of health: psychosocial adaptation

**DOI:** 10.15698/cst2024.03.294

**Published:** 2024-03-12

**Authors:** Carlos López-Otín, Guido Kroemer

**Affiliations:** 1 Centre de Recherche des Cordeliers, Equipe labellisée par la Ligue contre le cancer, Université Paris Cité, Sorbonne Université, Inserm U1138, Institut Universitaire de France, Paris, France.; 2 Facultad de Ciencias de la Vida y la Naturaleza, Universidad Nebrija, Madrid, Spain.; 3 Departamento de Bioquímica y Biología Molecular, Instituto Universitario de Oncología (IUOPA), Universidad de Oviedo.; 4 Metabolomics and Cell Biology Platforms, Institut Gustave Roussy, Villejuif, France.; 5 Institut du Cancer Paris CARPEM, Department of Biology, Hôpital Européen Georges Pompidou, AP-HP, Paris, France.

**Keywords:** mental health, aging, psychiatry, psychology

## Abstract

The eight biological hallmarks of health that we initially postulated (*Cell*. 2021 Jan 7;184(1):33-63) include features of spatial compartmentalization (integrity of barriers, containment of local perturbations), maintenance of homeostasis over time (recycling & turnover, integration of circuitries, rhythmic oscillations) and an array of adequate responses to stress (homeostatic resilience, hormetic regulation, repair & regeneration). These hallmarks affect all eight somatic strata of the human body (molecules, organelles, cells, supracellular units, organs, organ systems, systemic circuitries and meta-organism). Here we postulate that mental and socioeconomic factors must be added to this 8×8 matrix as an additional hallmark of health (“psychosocial adaptation”) and as an additional stratum (“psychosocial interactions”), hence building a 9×9 matrix. Potentially, perturbation of each of the somatic hallmarks and strata affects psychosocial factors and vice versa. Finally, we discuss the (patho)physiological bases of these interactions and their implications for mental health improvement.

## INTRODUCTION

The comprehension of health –defined in a positive fashion rather than as the absence of disease–requires a theory. We recently launched the bases of such a health theory by enumerating the fundamental biological characteristics or “hallmarks” of healthy organisms [1]. We concluded that the hallmarks of health include features of *spatial compartmentalization* (**Hallmark 1**: integrity of internal and external barriers; **Hallmark 2**: containment of local perturbations), *maintenance of homeostasis over time* (**Hallmark 3**: recycling & turnover of building blocks of biological systems; **Hallmark 4**: integration of circuitries; **Hallmark 5**: rhythmic oscillations with supra-, circa- and infradian periodicity), and *adequate responses to stress* (**Hallmark 6**: homeostatic resilience; **Hallmark 7**: hormetic regulation; **Hallmark 8**: repair & regeneration) **(Box1)**. We postulated that these eight hallmarks affected the organism through all eight organizational strata of the body including molecules, organelles, cells, supracellular units, organs, organ systems, systemic circuitries, and the meta-organism or holobiont [1].

BOX 1 **The molecular and cellular hallmarks of health**

Three years ago, we tentatively launched the modern bases of a theoretical explanation of health in which we didactically enumerated the fundamental biological characteristics or “hallmarks” of healthy organisms (López-Otín & Kroemer, *Hall-marks of Health*, Cell 2021). To qualify as a “hallmark” of health, we postulated that a process would have to fulfil three basic criteria, namely (i) invariably manifest in the context of sustained health, (ii) inexorably cause the loss of the healthy state, if perturbed or disrupted, and (iii) vigorously maintain or improve health, if experimentally accentuated or restored. When applying these stringent criteria, we finally defined eight molecular and cellular hallmarks of health classified in three categories:

**A. *Spatial compartmentalization***

**Hallmark 1**: integrity of internal and external biological barriers

**Hallmark 2**: containment of local perturbations in space and time

**B. *Maintenance of homeostasis over time***

**Hallmark 3**: recycling and turnover of all major building blocks of biological systems

**Hallmark 4**: integration of molecular, cellular, and long-distance communication circuits

**Hallmark 5**: rhythmic oscillations with supra-, circa- and infradian periodicity

**C. *Adequate responses to stress***

**Hallmark 6**: homeostatic resilience to maintain multiple biological parameters at adequate levels

**Hallmark 7**: hormetic regulation to acquire resilience against toxins and other stressors

**Hallmark 8**: repair and regeneration methods to sense and respond to the multiple body damages

**Figure 1:**
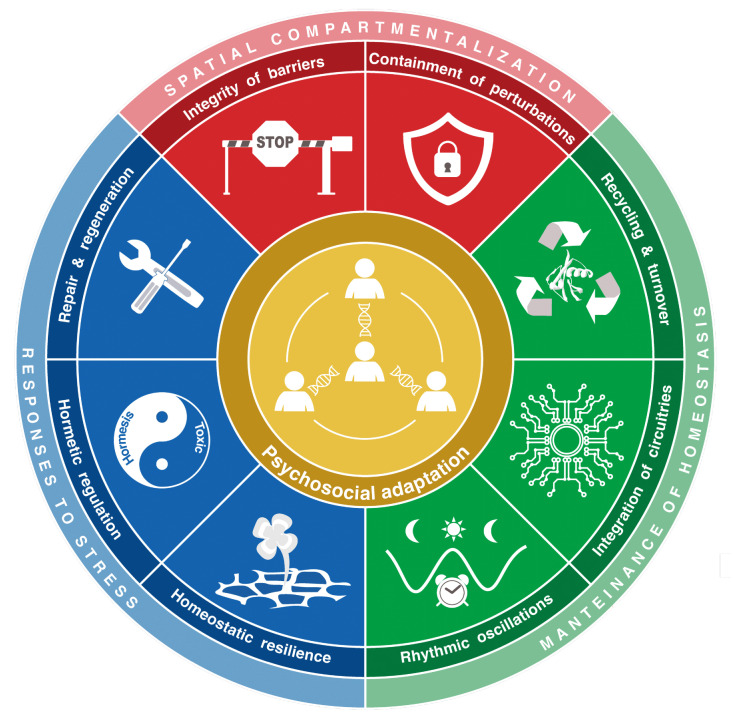
FIGURE 1: The psychosocial adaptation as a new hallmark of health. The scheme represents the links between psychosocial adaptation and the eight previously proposed hallmarks of health: integrity of barriers, containment of local perturbations, recycling & turnover, integration of circuitries, rhythmic oscillations, homeostatic resilience, hormetic regulation, and repair & regeneration. These hallmarks are grouped into three categories: spatial compartmentalization, maintenance of homeostasis over time, and adequate responses to stress.

**Figure 2:**
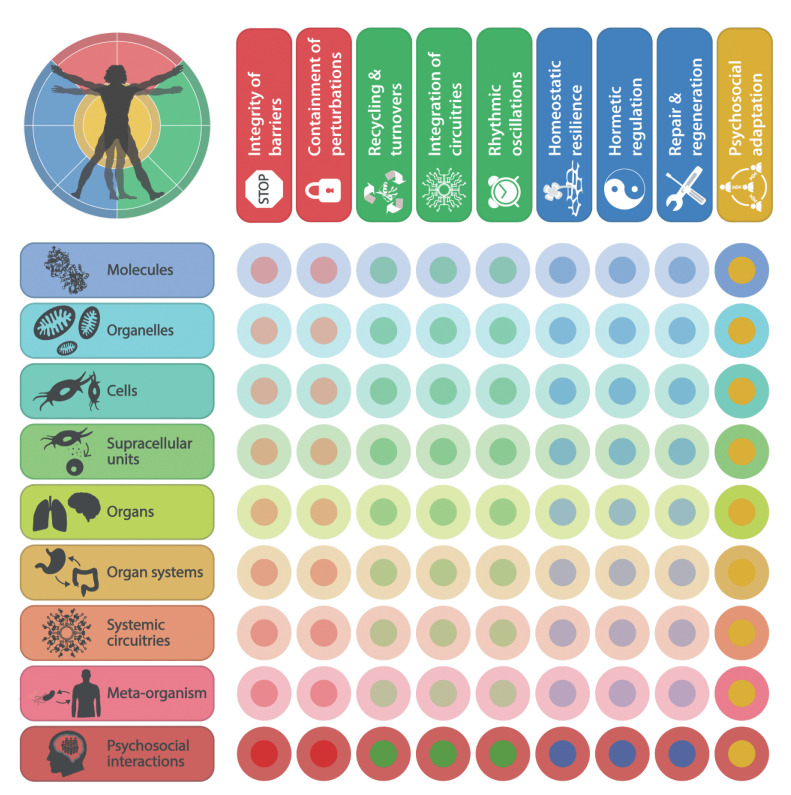
FIGURE 2: Square matrix representing the relationships between health hallmarks and body strata. The 9×9 matrix depicts the bidirectional connections between the nine hallmarks of health and the nine organizational strata of the human body. The relationships between psychosocial adaptation and psychosocial interactions are specifically enhanced.

In retrospect, we consider that our theory of health should incorporate one additional hallmark and one essential stratum, both of which are related to the psychosocial dimension of the human being. We postulate herein a ninth hallmark that we refer to as “psychosocial adaptation” **(Fig. 1)**, as well as a ninth organizational stratum (“psychosocial interactions”), hence extending the biological/somatic 8×8 matrix to a larger 9×9 matrix **(Fig. 2)**. In this article, we first define the ninth hallmark and evoke the impact of each of the eight biological hallmarks on mental health. Next, we show that perturbation of each of the somatic hallmarks and strata may affect psychosocial factors and vice versa. Finally, we discuss the (patho)physiological bases of these interactions and their potential applications for the improvement of mental health.

## PSYCHOSOCIAL ADAPTATION AS AN ESSENTIAL HALLMARK OF HEALTH

According to the World Health Organization (WHO), “mental health is a state of mental well-being that enables people to cope with the stresses of life, realize their abilities, learn well and work well, and contribute to their community”. In this sense, mental health is a larger concept than the mere absence of psychiatric disease. Currently, the classification of mental disorders presents several challenges including genetic overlaps between different conditions such as autistic spectrum disorders (ASD), major depressive disorder (MDD), bipolar disorder (BD) and schizophrenia (SCZ) [2, 3]. Such overlaps also involve alterations in brain anatomy, perturbations in gene expression signatures, symptomatic changes in mood and social behavior, and therapeutic responses to psychotropic drugs [4, 5]. In view of these uncertainties, the negative definition of mental health as the absence of mental disease appears imprecise.

The concept of “psychosocial adaptation” refers to the permanent tension between the individual and its social and socioeconomic context, which accompanies our personal trajectory from birth to death. This conflict has to be permanently resolved by adaptations that optimize the individual's capacity to cope with frustrations, to deal with the absence of positive social relationships, to avoid accidents and personal demolition, to successfully compete for resources and to contribute to the collective success of the social groups, while proactively taking the correct decisions [6]. Maladaptive reactions subvert subjective wellbeing and endanger the position and even the survival of the individual in the socioeconomic system [6], although sometimes the majority may take wrong decisions e.g. in a mass formation/mass psychosis situation. Our definition of psychosocial adaptation is not only circumscribed to the inner state of the individual and to the stressors that influence it. Previous studies have shown that positive social relationships are essential regulators of human physiology both in early and later life, as illustrated by works on maternal separation or maternal immune activation [7], and on interventions against the epidemic of loneliness [8]. Thus, psychosocial adaptation and development is not simply about the absence of social problems, but also requires the presence of stable and positive social interactions which represent critical sources of resilience to life stress [9–12]. Importantly, even extreme adversity does not necessarily undermine mental health [13]. While some individuals declare long-lasting mental problems after adverse experiences, others manage to cope with these events and maintain the state of “psychosocial adaptation”. This capacity is determined by genetic and non-genetic factors, as demonstrated by twin studies [14] and experiments on inbred mice [15], that have revealed interindividual differences between susceptibility and resistance/resilience to mental disease.

### Social Stress Models in Rodents

**Figure 3:**
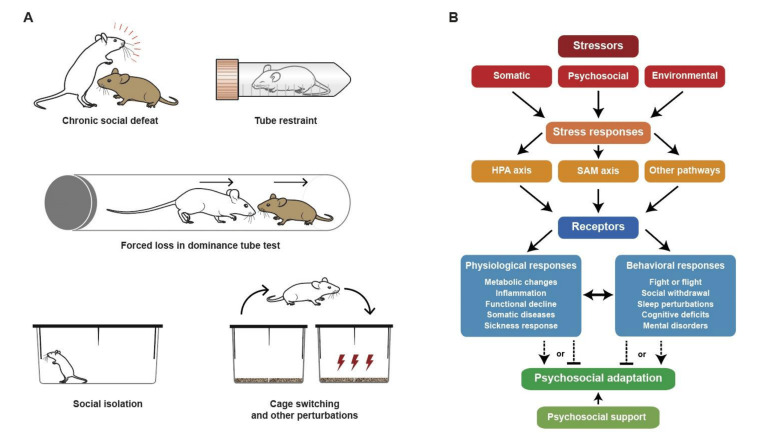
FIGURE 3: Mouse models of psychosocial stress and response pathways resulting in psychosocial adaptation. **(A)** Prototypic models of psychosocial stress in mice include chronic social defeat and dominance tube test models, as well as models of acute stress such as tube restrains, cage switching and short-term social isolation. **(B)** Different stressors including psychosocial stress promote a compendium of physiological and behavioral alterations coupled to activation of the sympathetic-adrenal medullary (SAM) and the hypothalamic pituitary adrenal (HPA) axes, as well as additional pathways which need to be further characterized. Deconvoluting the molecular pathways linking social stress to compromised mental and physical health may lead to the introduction of intervention strategies for improving psychosocial adaptation to stress.

A prototypic model of psychosocial stress in mice consists in the exposure of male mice to repeated aggression by male mice from an intruder strain **(Fig. 3A)**. This model of chronic social defeat promotes behavioral alterations coupled to an activation of the sympathetic-adrenal medullary (SAM) and hypothalamic pituitary adrenal (HPA) axes, which culminates with an increase in circulating catecholamines and glucocorticoids, and pro-inflammatory reactions that contribute to the behavioral phenotype due to neuroinflammation **(Fig. 3B)** [16]. Models of acute stress including tube restrains, cage switching, and short-term social isolation cause an increase in circulating interleukin-6 (IL6) levels secondary to SAM activation **(Fig. 3B)**. Then, IL6 stimulates hepatic gluconeogenesis and hyperglycemia to fuel the “fight or flight” response, but also increases the susceptibility to inflammation [17]. In yet another model of chronic isolation stress, HPA-independent brain-wide upregulation of tachykinin 2 (Tac2), a neuropeptide previously implicated in fear memory consolidation, induces enhanced aggression and other behavioral changes, which has suggested a role for Tac2 as an important mediator of the effects of chronic social isolation stress [18]. Social status among male mice can be studied in the dominance tube test **(Fig. 3A)** [19]. After repeated “forced loss” procedures (based on an experimental design in which a dominant mouse is repeatedly forced to back down and lose to a subordinate in the social hierarchy) the formerly dominant mouse loses its social rank and develops a depressive-like behavior coupled to activation of the lateral habenula. Likewise, loss of social status rather than a stable low rank constitutes a risk factor of MDD in primates including humans [20].

### Social and Socioeconomic Determinants of Health in Humans

Social stress increases the probability of developing multiple somatic and psychiatric diseases [6, 21]. Similarly, perceived social isolation correlates with enhanced severity of symptoms after viral immune challenge, inflammatory reactions, mental and physical morbidity, as well as with higher mortality rates [22]. Conversely, individuals exhibiting a high degree of social integration are afflicted by a relatively low morbimortality [23]. Differences in socioeconomic status may translate in discrepancies of a decade or more of disability-free life expectancy, coupled to a reduction of the prevalence of most major diseases in favor of the rich [6]. Similarly, the poor are more likely than the affluent to experience MDD and anxiety disorders [24]. The reasons for this association are multifold with higher chances of early life adverse events, hardship-induced stress, poor nutrition, less exercise, higher exposure to tobacco, alcohol and drug abuse, environmental pollution, extreme temperatures, violence and crime, as well as shame, emotional abuse, bullying, discrimination and isolation. In addition, poor somatic and mental health may predispose to low socioeconomic status through a “poverty trap” mechanism that includes reduced cognitive functions, lack of motivation and fatigue, as well as poor economic choices, suggesting a bilateral relationship between poor mental health and low socioeconomic status [24].

The temporal order of events observed in patients as well as preclinical experimentation suggest that prevalent characteristics linked to low socioeconomic status may contribute to mental illness, as documented for obesity, which is associated with reduced cognitive functions in adults [25] and suppresses neurogenesis and causes anxiety in mice, as well as increased susceptibility to several mental pathologies including depression, psychosis, anxiety, and eating disorders in human [24, 26]. Most current evidence, in particular that drawn from animal models, support the “social causation” hypothesis, meaning that social interactions directly affect health outcomes [6].

### Psychosocial Adaptation for the Improvement of Health

Loss of psychosocial adaptation often occurs as a correlate of deteriorating health conditions and interventions favoring this adaptation improve health outcomes in clinical trials [27–30]. Further, psychosocial interventions increase stress resilience [31] and improve the recovery from depression [32]. Several studies have suggested that inhibition of stress mediators such as inflammatory cytokines, glucocorticoids and catecholamines elicited by social stress can mediate effects on psychiatric diseases [33–37], while other works have shown that the presence of mental disorders amplifies morbidity or mortality due to somatic disease [38]. Accordingly, treatment of mental disorders with psychotropic drugs does not only reduce psychiatric symptoms, but also mitigates excessive somatic morbimortality [39].

In summary, the enfeeblement of somatic health impacts psychosocial adaptation and vice versa. Emerging evidence suggests that treatments designed to enhance psychosocial adaptation, to improve stress management, or to provide adequate psychopharmacological care, may have a positive impact on health outcomes. Based on this information, we aimed to unveil the connections between psychosocial adaptation and somatic hallmarks of health.

## HALLMARK 1: INTEGRITY OF BARRIERS

**Figure 4:**
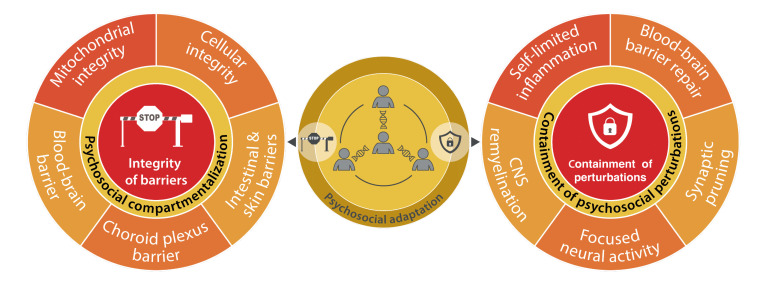
FIGURE 4: Psychosocial dimension of health hallmarks implicated in spatial compartmentalization. Links between psychosocial adaptation and the mechanisms responsible for the integrity of biological barriers and the containment of local perturbations.

The first hallmark of health consists in the integrity of biological barriers [1]. Such barriers must maintain strict compartmentalization to ensure the functional organization of the organism, but allow for the controlled exchange of solutes, electrolytes, soluble factors and mobile cells to sustain health. Here, we will examine how mental disorders compromise the integrity of barriers and how this integrity may impact psychosocial parameters **(Fig. 4)**.

### Mitochondrial Integrity

Mitochondrial structure determined by fusion/fission events as well as the expression of bioenergetically relevant pumps and enzymes determine the propensity of mitochondria to undergo permeabilization and hence to release pro-inflammmatory and pro-apoptotic molecules into the cytosol of stressed cells. Mitochondrial DNA (mtDNA) alterations are associated with human ASD [40] and autism-like phenotypes in mice [41]. Induced pluripotent stem cell (iPSC)-derived neural precursors from BD patients present a mitochondrial defect which can be reversed by lithium [42]. MDD has been associated with increased circulating levels of acylcarnitines – suggesting a defect in mitochondrial β-oxidation – [43] and of cell-free mtDNA derived from mitochondrial membrane permeabilization [44]. Notably, the 22q11.2 deletion syndrome, which often includes facets of ASD and SCZ [45], leads to mitochondrial disruption in mouse cortical neurons [46]. iPSC-derived neurons from 22q11.2-deleted patients with signs of SCZ manifest a defect in oxidative phosphorylation [47], while transplantation of normal mitochondria into the prefrontal cortex of adolescent rats suppresses SCZ phenotypes induced by maternal immune activation [48], further supporting the relationship between SCZ and mitochondrial dysfunction.

### Cellular Integrity

In a mouse model of depression induced by chronic mild stress, astrocytic pyroptosis (an inflammatory form of cell death triggered by microbial infections and host factors) may contribute to this condition since knockout of pro-pyroptotic genes alleviates depression-like behavior [49]. Cell-free genomic DNA is increased in plasma from SCZ patients, supporting the possibility of increased cell death [50]. Brain-derived cell-free DNA, detected via epigenetic markers, is increased in SCZ patients with first psychotic episodes [51]. This may represent a sign of enhanced cell death by plasma membrane permeabilization, reduced clearance of dead cells, or deficient retention of cellular debris by the blood-brain barrier (BBB) in the central nervous system (CNS) from these patients. However, the literature on excessive cell death in mental disease is scarce, thus pointing to a higher importance of BBB integrity.

### Blood-Brain Barrier Integrity

BBB assures the exclusion of mobile immune cells and pro-inflammatory factors, the transport mechanisms from the periphery into the brain and the active export of toxic metabolites and waste products from the brain [52]. Endothelial cells, which form the first barrier between blood and the brain, are connected by tight junction proteins such as claudin-5 (CLDN5). The local abundance of this protein is often a proxy to assess BBB intactness, which is perturbed in aging and psychiatric diseases [52]. Notably, the 22q11.2-deletion syndrome causes *CLDN5* haploinsufficiency and leads to reduced CLDN5 levels in endothelial cells. In parietal lobes from non-syndromic SCZ patients, *CLDN5* is discontinuously expressed in the BBB [53], while reduced expression of *CLDN5* in the hippocampus of MDD or SCZ patients correlates with early onset and prolonged duration of disease [54]. Importantly, *CLDN5* expression in specific brain areas correlates with susceptibility or resistance to stress-induced disorders [55, 56], supporting the implication of the CLDN5-dependent BBB integrity in mental health maintenance. Accordingly, chronic social defeat stress in mice causes *Cldn5* downregulation in the nucleus accumbens with loss of BBB integrity [57]. This process is mediated by activation of TNFα/NFκB signaling and histone deacetylase 1 (HDAC1, that catalyzes the deacetylation of lysine residues of core histones), and by upregulation of the transcription factor forkhead box protein O1 (FOXO1) [58]. Of note, *HDAC1* and *FOXO1* are upregulated in the nucleus accumbens from untreated MDD patients, while *CLDN5* is downregulated [58]. Similarly, female MDD patients who died from suicide present downregulation of *CLDN5* in the prefrontal cortex [55]. Gain- and loss-of-function experiments in mice have provided causal support to the idea that BBB permeabilization is broadly neuropathogenic [55–57, 59]. Moreover, subtle alterations in BBB function that affect specific transport systems have been detected in neuropsychiatric patients [60, 61].

### Choroid Plexus Barrier Integrity

The choroid plexus vascular barrier, which separates blood from cerebrospinal fluid, is usually permeable to molecules of up to 70 kDa, yet closes upon induction of intestinal inflammation due to sealing of the fenestration of endothelial cells. Genetically-driven closure of the choroid plexus induces anxiety-like behavior that was also observed in intestinal inflammation, suggesting a pathogenic role for these alterations that potentially link inflammatory bowel disease and psychosocial disturbances [62]. In both MDD and psychosis patients, the choroid plexus is enlarged compared to healthy controls, and this finding correlates with signs of neuroinflammation [63]. Moreover, the choroid plexus is histologically altered in SCZ patients [64]. However, it remains to be determined whether these macro- and microscopic alterations reflect changes in barrier function.

### Intestinal Barrier Integrity

The gut vascular barrier protects from external insults through a multilayered structure that evolves in cooperation with the local microbiota. Numerous studies have suggested that general features of the unhealthy gut microbiota (dysbiosis) are non-specifically associated with multiple distinct disease states ranging through the entire spectrum of pathologies including oncological, metabolic, cardiovascular, and neuropsychiatric diseases. Hence, gut health is a common trait of general health. Accordingly, breaches in the mucus layer and the enterocyte epithelium [65] can lead to the translocation of microbes or microbe-derived molecules into the host, elicitation of inflammatory signals, and trafficking of gut-resident immune cells to other organs [66, 67]. Accordingly, “leaky gut” may trigger or modulate distinct diseases including mental disorders [66]. Psychological stress causing the activation of HPA or SAM impacts the gut. Thus, HPA-elicited chronic elevations in glucocorticoids have multiple effects on the gut due to the expansion of an inflammatory subset of enteric glia and the inhibition of acetylcholine responses by enteric neurons causing intestinal dysmotility [68]. In contrast, SAM activation accounts for a “stress ileopathy” with consequent shifts in the microbiota [69]. Reciprocally, the microbiota participates in HPA modulation, because its depletion exacerbates HPA activation in response to moderate stress [70]. Moreover, elevated levels of biomarkers of intestinal barrier permeability have been detected in patients with mood disorders [71]. Interestingly, fecal microbiota transplantation from MDD patients into rats induces a depressive-like phenotype [72], while lithium administration significantly increases species richness and diversity in the rat gut, likely contributing to the beneficial effects of this drug [73]. In mice, maternal immune activation or infection during pregnancy induces the differentiation of IL17–producing TH17 lymphocytes in the gut. IL17 then crosses the placental barrier and affects the fetal CNS, resulting in ASD-like behavior [74]. In this model, gavage with *Lactobacillus reuteri* corrects social impairments by signaling across the microbiota-gut-brain axis through vagal neurons [75]. In ASD patients, major shifts in the intestinal microbiota have been detected, but many of them are likely caused by altered food preferences of these patients [76]. Nonetheless, preclinical experimentation in mice indicates that bacterial L-tyrosine metabolites can induce anxiety-like behavior [77]. Moreover, an oral small-molecule sequestrant with affinity for microbiota metabolites mitigates anxiety and irritability in adolescents with ASD [78]. Altogether, it appears that some microbial metabolites (psychobiotics) can induce systemic effects on mental health. Such effects can be negative, as illustrated for ASD, but can also be positive, as exemplified by the microbiota-derived endocannabinoids that increase the motivation for physical exercise [79].

### Skin Barrier Integrity

The common manifestation of atopic dermatitis or psoriasis-two conditions with compromised skin barrier integrity- and different mental disorders might reflect common genetic causes simultaneously affecting the two tissues of ectodermal origin, skin and brain [80]. Independently of this speculative explanation, eczema and related atopic diseases are associated with more severe ASD manifestations. Hence, a hypothetical “skin-brain axis” has been proposed in which dermal inflammation would trigger mental disorders. Supporting this conjecture, experimental induction of dermatitis in mice increased anxiety- and depressive-like behaviors, along with elevated serum corticosterone levels [81]. More convincingly, in clinical trials, anti-IL17A [82] and anti-IL4R [83] antibodies which target inflammatory signaling in the skin, reduced psoriatic lesions and atopic dermatitis, respectively, as they simultaneously mitigated anxiety and depression.

## HALLMARK 2: CONTAINMENT OF LOCAL PERTURBATIONS

Perturbations due to endogenous alterations, infectious agents and mechanical, chemical, physical or emotional trauma can cause focal damage to tissues and compromise barriers. Failure to confine such perturbations, avoiding their spread to a systemic level and their perpetuation, is intrinsically pathogenic. Hence, the containment of local perturbations is essential for the maintenance of somatic and mental health **(Fig. 4)**. This is particularly well documented for inflammation, usually a local phenomenon that resolves. However, inflammation becomes broadly pathogenic if it acquires a system-wide, chronic dimension [1].

### Self-limited Inflammation

Neuroinflammation is linked to systemic inflammation and likely contributes to the pathogenesis of mental disorders [84]. Orthopedic surgery performed in older adults often induces delirium as a result of systemic inflammation spurring neuroinflammation. Postoperative delirium can be reduced in patients by the α2-adrenergic receptor agonist dexmedetomidine [85] and in mice by inhibition of microgliosis by ω-3 fatty acids or resolvins [86]. CNS-specific inflammation due to autoimmunity or stroke can trigger MDD. Thus, relapsing multiple sclerosis is associated with depression that responds to fingolimod [87], an immunosuppressor which is also active against SCZ [88]. Anti-TNFα-based treatments mitigate depressive symptoms in rheumatic diseases patients [89]. Likewise, intracerebroventricular infusions of anti-TNFα antibody or several resolvins produce antidepressant-like effects in rodent models [90], while in MDD patients, oral supplementation with the ω-3 fatty acid eicosapentaenoic acid induces clinical responses associated with elevated plasma concentrations of pro-resolving lipid mediators [91]. These findings support the possibility to intervene on neuroinflammation in mental disorders. If inflammation resolves in a timely fashion after an acute phase, it facilitates tissue repair [92]. In the CNS, such repair reactions allow to restore BBB integrity and axon remyelination which are essential for the maintenance of CNS function.

### Repair of the Blood-Brain Barrier

BBB permeabilization can be repaired through a process that involves the contribution of endothelial cells, pericytes, astrocytes and fibroblasts. BBB repair slows with aging [93], and can be stimulated by CNS-targeted gene therapy with engineered Wnt ligands [94] or by intracerebroventricular administration of protease inhibitors [95]. Traumatic brain injury in mice induces BBB permeabilization and axonal degeneration leading to progressive neurological and mental deficits. These long-term consequences of brain trauma can be prevented by activation of NAD^+^ biosynthesis by P7C3, an activator of NAMPT, and the NAD^+^ precursor nicotinamide riboside improved depressive- and anxiety-like behaviors in rats exposed to mild stress [96]. Similarly, nicotinamide, another NAD^+^ precursor, mitigates BBB damage and psychosis in rats chronically exposed to ketamine [97]. These observations suggest the use of NAD^+^ precursors in neuropsychiatric diseases linked to BBB permeabilization.

### CNS Remyelination

Human SCZ is accompanied by reduced myelination of the medial frontal regions. In rodents, demyelination resulting in SCZ-like behaviors can be induced by administration of cuprizone, a copper chelator that paradoxically increases copper concentrations in the brain [98]. Remyelination can occur spontaneously after cuprizone withdrawal but is enhanced by some antipsychotics and by the histamine receptor H1 antagonist clemastine [98]. Of note, clemastine can reduce depressive-like behaviors in mice exposed to social defeat stress [99]. Interestingly, clemastine has other pharmacological effects (e.g., anticholinergic) that increase the activity of histone methyltransferases and may also contribute to the behavioral benefits of this drug. Clemastine and sobetirom - another promyelinating drug - also promote functional recovery in an ASD mouse model [100]. Multiple remyelination-inducing drugs are being developed, mostly for multiple sclerosis treatment, and it will be interesting to learn whether they mediate positive effects on patients with mental disorders [101].

### Synaptic Pruning

Synaptic pruning is a fundamental neurodevelopmental process in which excess or weak synapses are eliminated to optimize neural circuitry [102]. Synaptic pruning also plays a crucial role in shaping circuits involved in learning and memory [103]. Both excessive and deficient synaptic pruning might contribute to mental disorder pathogenesis. Deficient pruning mechanisms during early brain development may result in atypical neural connectivity patterns, linked to the social and behavioral deficits observed in ASD patients. As compared to neurotypical controls, the frontal, temporal and parietal lobes from these patients exhibit increased synapse density, suggesting an underpruning phenotype [104]. Abnormalities in synaptic pruning have also been implicated in SCZ pathogenesis, which is associated with synaptic alterations suggestive of overpruning [105]. Dysregulated synaptic pruning during adolescence may underlie these structural changes and contribute to the cognitive deficits observed in SCZ [106]. Accordingly, SCZ is linked to allelic variants of the complement component 4A (*C4A)* locus that enhance C4a protein production, while overexpression of human *C4A* in mice causes excessive pruning in the cortex associated to SCZ-like behaviors [107].

## HALLMARK 3: RECYCLING AND TURNOVER

**Figure 5:**
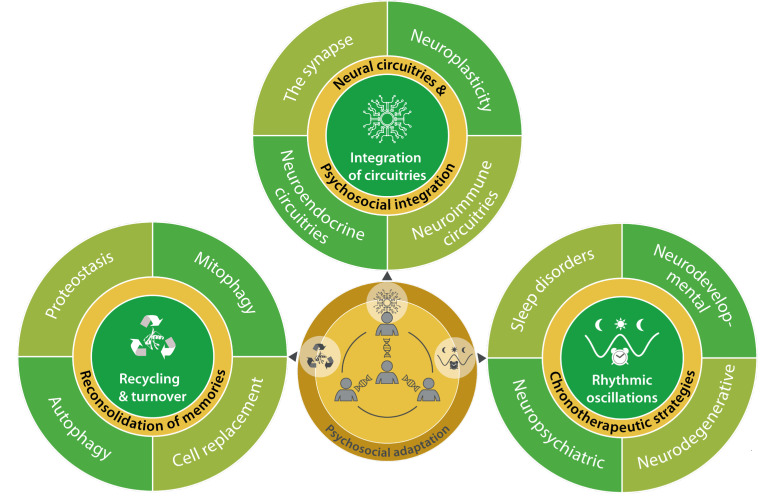
FIGURE 5: Psychosocial dimension of the hallmarks of health involved in maintenance of homeostasis. Interconnectivity between psychosocial adaptation and mechanisms of recycling and turnover in tissues and cells, crosstalk among different circuitries (cell-tissue-organ-system), and their synchronization with circadian, infradian or ultradian rhythmic oscillations.

Many of the building blocks of the organisms undergo spontaneous or stress-induced alterations that must be counterbalanced by their constant dismantling and rebuilding. Recycling and turnover hence are critical for the maintenance of the healthy state, as this is particular well documented for protein homeostasis (proteostasis), autophagy and cell replacement [1]. Alterations in these processes are also associated with mental illnesses **(Fig. 5)**.

### Proteostasis

Aberrant proteostasis is a hallmark of aging and neurodegenerative diseases [108, 109]. Proteostasis alterations in neuropsychiatric disorders do not result in massive neuronal death, but in diverse loss- and gain-of-function events converging in the disruption of synaptic and neural functions [110–112]. Specific proteases and E3 ubiquitin ligases are mutated in hereditary mental and neurodevelopmental disorders [113–117]. Conversely, loss of ErbB3-binding protein 1 (EBP1, a signaling molecule which is mutated in some SCZ patients) causes upregulation of the E3 ubiquitin ligase Fbxw7 and an SCZ-like behavior in mice [118]. Moreover, in fragile X syndrome – a leading monogenic cause of autism – loss of Fragile X Mental Retardation protein 1 (FMRP1) impairs proteostasis. This deficiency can be mitigated in mice by administration of proteasome inhibitors, which also attenuate hyperexcitability in response to auditory stimulation [119]. These findings point to the causal involvement of specific alterations of proteostasis in neuropsychiatric diseases.

### Mitophagy

Mitochondrion-specific autophagy (mitophagy) involves several genes/proteins that mark dysfunctional mitochondria for autophagic destruction and hence serve as “autophagy adaptors” – such as *PARK* and *PINK* – that are mutated in Parkinson's disease patients [120]. Of note, Parkinson's disease is not limited to motor deficits, but is usually preceded and accompanied by neuropsychiatric alterations and cognitive impairment [121]. Disrupted-in-SCZ-1 (DISC1) – the mutation of which causes behavioral abnormalities – has been identified as a mitophagy receptor [122]. Defective mitophagy might also contribute to MDD-associated mitochondrial dysfunction [123]. In contrast, excessive Pink- and Park-dependent mitophagy in the basolateral amygdala from mice has been proposed to mediate disproportionate elimination of mitochondria in chronic social defeat stress, facilitating anxiety and aversive social behavior [124]. Hence, both deficient and excessive mitophagy may contribute to the pathogenesis of mental disorders.

### Autophagy

Macroautophagy (to which we refer to as autophagy) can be activated in all relevant cell types of the CNS [125]. Neuron-specific knockout of the essential autophagy gene *Atg5* results in increased excitatory neurotransmission [126]. Autophagy also contributes to synaptic remodeling, for instance in long-term synaptic depression induced by NMDAR activation [127]. In microglia, knockout of another essential autophagy gene, *Atg7*, impairs synaptic pruning and elicits ASD-like behaviors [128]. Moreover, genetic defects in specific autophagy genes cause neuropsychiatric disorders [125, 129, 130]. *Tsc2*^+/-^ mice -which manifest constitutive overactivation of nutrient sensor mTorc1 and tonic inhibition of autophagy-exhibit ASD-like features [131]. Similarly, valproic acid administration to mice and vitamin B6 deficiency in rats are associated with autism-like behavior that can be reversed by mTorc1 inhibition with rapamycin [132, 133]. Obesity activates mTORC1 in microglia and astrocytes with disabled autophagy, while rapamycin treatment restores autophagy and reduces depressive and anxiety-like behaviors [134]. Autophagy inhibition caused by obesity might explain the link between excessive adiposity and ASD, MDD or SCZ [135, 136], but the observations suggesting that fasting may improve MDD must be replicated in large randomized studies [137]. These findings suggest that mTORC1 is hyperactivated in neuropsychiatric diseases, but there are exceptions to this rule. Thus, mTORC1 hypoactivity in the prefrontal cortex from men with BD and psychosis is pathogenic [138]. Accordingly, the antidepressant actions of ketamine depend on the neural activation of mTORC1 and its downstream effectors [139]. Moreover, systemic Gdf11 injection into aged mice alleviates depression-like symptoms through mTorc1 activation in hippocampal neurons [140]. Likewise, some of the neuroprotective mechanisms induced by lithium in BD and other neuropsychiatric conditions may be related to autophagy regulation, although the underlying mechanisms are still unclear.

Common antidepressants induce autophagy in circulating leukocytes from MDD patients [141]. as well as in mouse hippocampal neurons secondary to the accumulation of ceramide in the endoplasmic reticulum and sphingomyelin in lysosomes and Golgi apparatus [142]. Direct inhibition of sphingomyelin synthase with D609 enhances accumulation of ceramide and activation of autophagy, and reduces stress-induced MDD. Moreover, mice lacking acidic sphingomyelinase – which converts sphingomyelin into ceramide – exhibit depressive behavior, while the antidepressant effects of amitriptyline and D609 are abolished by the autophagy inhibitor spautin [142]. These findings suggest that antidepressants may exert some of their beneficial effects through the enhancement of autophagic flux.

### Cell Replacement

Cell death requires a local response for efficient corpse removal by phagocytes, and for replacing the missing cell or palliating its absence. Neuronal cell death triggers an orchestrated reaction by astrocytes and microglia, which engulf dendritic arbors, and the soma from neurons, respectively [143]. In the CNS, different cell types undergo replacement at rather different rates. While most neurons are post-mitotic cells that should finish their existence when their host dies, there is evidence for postnatal neurogenesis in some human brain areas, and impaired neurogenesis has been involved in the pathogenesis of several neuropsychiatric conditions (see Hallmark 8).

Ablation of brain astrocytes can induce a compensatory proliferation of neighboring juxtavascular astrocytes [144]. Nerve growth factor receptor (p75NTR)-dependent astrocyte proliferation has also been observed after brain injury [145]. In contrast, astrocyte-specific knockout of *Soc2* interferes with astrocyte proliferation and improves recovery from traumatic brain injury, yet causes a hyperactivity/hyperexcitability phenotype [146]. Hence, the role of astrocyte proliferation and differentiation in brain health requires further investigation. Microglial progenitor cells formed in the embryonic yolk sac enter the CNS before the BBB forms and then constitute a self-renewing population without further contribution by the hematopoietic system [147]. With age, microglia is characterized by an increase in senescent cells coupled to the activation of the pro-inflammatory senescence-associated secretory phenotype. Chemogenetic ablation and systemic senolysis of such senescent microglial cells improves cognition in aged mice [93], while forced turnover of microglia following colony stimulating factor 1 receptor (CSF1R) inhibition improves recovery from traumatic brain injury at the inflammatory and neuropsychiatric levels [148]. These results suggest that strategies for increasing microglial renewal might be useful for intervening on neuropsychiatric diseases.

## HALLMARK 4: INTEGRATION OF CIRCUITRIES

The organism – including the nervous system – is built in a way that molecular, intracellular, extracellular and long-distance neuroendocrine communication systems constitute interwoven circuitries, favoring integration [1]. We posit that loss of integration is invariably pathogenic, including at the psychosocial level **(Fig. 5)**.

### The Synapse

Numerous genes whose mutations or variations are involved in mental diseases encode proteins acting at the level of synapses [149]. Such genes may also be modulated epigenetically by early-life experiences, environmental factors and stress [149]. A few examples underscore the implication of synaptic alterations in mental disorders. A mutation in the cell adhesion protein neuroligin-3 (NLGN3) linked to ASD induces an enhancement in excitatory synaptic transmission [150], while loss of synapses in SCZ disrupts pyramidal neuron function in the cortex to elicit cognitive symptoms and disinhibits mesostriatal projections to promote dopamine overactivity and psychosis [106]. In response to prolonged stress, prefrontal cortex and hippocampus undergo dendritic retraction and spine loss, while other regions including amygdala and lateral habenula manifest elevated spine density and potentiated activity [151]. Clinically active antidepressants reverse the effects of stress and depression on synapse function, augmenting neurotransmission, boosting plasticity, and favoring synaptogenesis [151].

### Neuroplasticity

CNS is endowed with an unmatched capacity to simultaneously capture sensory, emotional and cognitive inputs and to stock, encrypt, retrieve and utilize information through an integrative learning process compatible with creativity, imagination and improvisation [152]. Neuroplasticity is achieved by a combination of three mechanisms. First, synaptic plasticity is shaped by coordinated neuronal activity, reinvigorating the strength and efficiency of synaptic connections by long-term potentiation or attenuating their function by long-term depression [153]. Second, structural plasticity involves reorganization of interneuronal connections by the pruning of dendritic spines [154]. Third, neurogenesis facilitates the generation of new neurons in specific brain regions. Beyond its role in neurodevelopment and the acquisition of specific talents, neuroplasticity is crucial in the brain's adaptive capacity to reorganize and compensate for deficits caused by stroke, trauma or sensory deprivation. Neuroplasticity can also be maladaptive in specific neurological conditions such as phantom limb sensations, chronic pain, hyperactivity and addiction. Maladaptive neuroplasticity can be tackled by several strategies including transcranial stimulation methods and biofeedback techniques [155]. In addition, ketamine and psychedelics may mediate their antidepressant effects by stimulating neuroplasticity [156].

### Neuroendocrine Circuitries and Interoception

The nervous and endocrine systems are linked through intricate bidirectional interactions. Starting by neural inputs, these circuitries involve SAM and HPA, but also thyroid and sex hormones, and the pro-social hormone oxytocin [157, 158]. Conversely, stress hormones, classical hormones produced by endocrine organs and a long list of “tissue hormones” impact the CNS. These tissue hormones include adiponectin, which has neuroprotective effects but also increases susceptibility to social stress;[159]. diazepam-binding inhibitor, which stimulates central appetite centers but may also cause depression-like behavior;[160]. and glucagon-like peptide-1, which induces satiety but may also attenuate depression [161]. These examples illustrate a constant neuroendocrine communication between CNS and the endocrine system that allows for the coordination of mental and bodily functions.

Interoception permanently informs the brain on multiple physiological parameters to generate a representation of the internal state of the organism and to facilitate adequate reactive or proactive control [162]. Cardiac and gastric interoception involves parasympathetic and sympathetic signals, as well as subcortical relay nuclei including the nucleus tractus solitarius and parabrachial nucleus [163]. Dysfunctional interoception may compromise mental health and participate in the pathogenesis of anxiety and mood and eating disorders [164]. Of note, optogenetically induced extreme tachycardia causes anxiety in mice via activation of the posterior insular cortex [165], demonstrating that peripheral organs responding to stress may control the affective behavioral state, hence closing an anxiogenic feedforward loop [165]. The disruption of such a malicious circuitry may contribute to the anxiolytic and antidepressant effects of calcium channel blockers and β-adrenergic receptor antagonists used for the treatment of tachycardia and hypertension [166].

### Neuroimmune Circuitries

Cytokines released by immune cells in response to pathogens may act as interoceptive signals since they transmit signals across BBB, including stimulation of the afferent (sensory) vagus nerve. This elicits a reciprocal response via HPA. In addition, the CNS can stimulate the efferent vagus nerve of the parasympathetic nervous system to exert a systemic anti-inflammatory effect termed as the “inflammatory reflex”. Signals communicated via the vagus and splenic nerves cause T cells to produce acetylcholine that acts on macrophages to dampen inflammation [167]. Disruption and mimicry of this reflex may exacerbate or suppress inflammation and depression [167]. Genetic models of immunodeficiency have supported the link between the cellular immune system and anxiety-like behaviors [168]. T cells can affect behavior in multiple ways. For example, adoptive transfer of CD4^+^ T lymphocytes from stressed mice to non-stressed recipients induces anxiety-like behavior [169]. In the chronic social defeat model, susceptible mice manifest the depletion of *Lactobacillus johnsonii* from their gut microbiota coupled to an increase in the frequency of IL17-producing γδ T cells in the colonic lamina propria, as well as in the meninges. In this model, supplementation with *L. johnsonii* or depletion of γδ T cells suppresses social avoidance [170]. Collectively, these observations suggest the existence of multiple yet-to-be-discovered circuitries connecting the brain to the peripheral immune system.

## HALLMARK 5: RHYTHMIC OSCILLATIONS

Biological clocks establish the rhythms of life that orchestrate the complex mechanisms underlying organismal homeostasis, including those necessary for the maintenance of mental health [171]. The central component of this synchronization system is a circadian clock located in the suprachiasmatic nucleus of the hypothalamus. These neural pacemakers receive information on light cues from photoreceptive retinal cells and specialized retinal ganglion cells, and then confer circadian rhythmicity to the myriad of peripheral clocks present in the diverse body tissues [172]. Circadian oscillations are molecularly driven by intricate transcriptional-translational feedback loops involving the transcriptional activators BMAL1 and CLOCK, which transactivate the genes encoding cryptochromes CRY1 and CRY2, and the transcriptional repressors PER1 to PER3, which in turn inhibit *BMAL1* and *CLOCK* expression [172]. Disruption of circadian rhythms due to alterations in core clock genes or to lifestyle changes is responsible for a variety of human pathologies -including sleep disorders, neurodevelopmental conditions, and neurodegenerative processes- which compromise mental health and interfere with social relationships [173] **(Fig. 5).**

### Sleep Disorders

Circadian misalignment of environmental cues with the endogenous clock program due to artificial lighting, shift work or jet travel causes sleep disorders and is broadly pathogenic. Blue light that is continuously emitted by electronic devices used in daily life shifts the phase of neuronal and peripheral-tissue clocks [174]. Likewise, work scheduled during normal sleep time or frequent traveling across time zones desynchronizes sleep-wake rhythms from the light-dark natural cycle and leads to excessive sleepiness or insomnia. Additionally, intrinsic alterations of circadian function result in heritable early or late chronotypes characterized by an extremely advanced or delayed onset of sleep, which may impact on physical and cognitive performance, but also on mood status and social interactions [175]. Sleep disturbances together with inadequate eating schedules contribute to misalign clocks in metabolic organs, leading to obesity and other metabolic disorders, which in turn amplify mental perturbations. The early identification of individuals with extreme chronotypes, which are at risk of sleep dysfunctions, as well as behavioral and pharmacological interventions can reinforce circadian rhythmicity and improve the control of sleep-wake cycles [176].

The relevance of sleep timing and chronotypes for the maintenance of mental health has grown in the context of “social jetlag” [177]. This term defines the discrepancy between biological time, dictated by internal circadian clocks, and social time, determined by social activities. Epidemiological studies have identified associations between social jetlag and the prevalence and clinical onset of diverse disorders, ranging from depression to metabolic dysfunction and reduced cognition [178–180]. Accordingly, the social zeitgeber theory proposes that disruption in the timing of daily social routines increases the risk for mood disorders and exacerbates BD [181]. Hence, social jetlag may be considered as a public health risk.

### Neurodevelopmental and Neuropsychiatric Diseases

Circadian clock disruption has been detected in psychiatric disorders characterized by temporal or seasonal changes [182]. Such disorders are commonly associated with alterations in different biological rhythms, including those controlling the sleep-wake cycle, cortisol and melatonin production, blood pressure, and circadian variations in the expression of clock genes and their transcriptional targets. Such changes occur across a large spectrum of neuropsychiatric disorders, likely contributing to their overlapping symptoms [183]. Genetic studies have identified circadian clock gene variants associated to mood disorders, although data are not univocal, probably due to the variability of environmental influences in diverse study populations [173]. Neuroimaging analysis has suggested that infradian fluctuations in the sensorimotor network and in subcortical 5-hydroxytryptamine projection regions explain the seasonality of psychiatric diseases [184]. Neurodevelopmental syndromes such as Prader-Willi syndrome are also associated with dysfunctional circadian rhythms [185], while studies with mutant mice deficient in clock genes have confirmed that the circadian clock modulates mood-related behaviors [186, 187]. Several molecular pathways (i.e., the HPA axis and monoaminergic neurotransmission) are involved in the circadian clock disruption observed in SCZ and mood disorders in preclinical models [188–190]. Substance abuse disorders are also associated with desynchronization of circadian rhythms occurring during the transition from recreational consumption to addictive behavior [191]. This process involves the activation of the dopamine D2 receptor, which then triggers a regulatory circuit that finally leads to the activation of the PPARγ nuclear receptor. Interestingly, administration of pioglitazone, a specific PPARγ agonist to *D2r*-deficient mice restores adequate rhythms of circadian genes [191], pointing to new opportunities for the treatment of drug addiction disorders.

### Neurodegenerative Diseases

Experimental jet lag in mice inhibits adult neurogenesis and causes cognitive impairments [192]. The circadian clock is functionally disrupted in patients with different neurodegenerative disorders, such as Alzheimer's, Huntington's and Parkinson's diseases, which in turn are linked to deficient adult neurogenesis. Mammalian clock genes participate in the control of neurogenesis by restricting the expansion of rapidly dividing neural precursors and by regulating the entry of quiescent neural stem cells into the cell cycle. Circadian rhythms in neural stem cells are regulated by glucocorticoids through a balanced action on mineralocorticoid and glucocorticoid receptors. Mice deficient in clock genes lack the circadian gating of cell cycle and lose diurnal rhythmicity [192]. Likewise, patients with neurodegenerative diseases often exhibit a severe reduction in the robustness of the circadian clock that results in profound disturbances of sleep–wake cycles [193]. Moreover, polymorphisms in clock genes have been associated with an increased risk of Alzheimer's or Parkinson's disease, while preclinical and clinical data have correlated circadian disruption with the accumulation of neurotoxic proteins and neurodegeneration itself [173, 182]. Finally, the lack of appropriate light-dark cues, the presence of irregular sleep-wake cycles and the functional deterioration of circadian clocks contribute to the “sundown syndrome” which is prevalent in people with dementia or neurodegenerative illnesses.

All these circadian system-related alterations have been traditionally viewed as correlative rather than causal events, but recent studies indicate that signs of circadian disruption precede the manifestation of other clinical symptoms, reinforcing the idea that perturbation of biological rhythms contribute to disease pathogenesis [194]. Accordingly, chronotherapeutic interventions aimed at resynchronization of these rhythms have shown promising effects [183]. An illustrative example is the antidepressant agomelatine which directly targets the circadian system, acting as a melatonin-receptor agonist and also as a 5-hydroxytryptamine 2B/2C receptors antagonist. Agomelatine resynchronizes disrupted circadian rhythms and improves sleep patterns in patients with autism, attention-deficit/hyperactivity disorder, anxiety, and depression [195]. Therefore, circadian medicine and chronotherapy – which target specific clock components while carefully timing the administration of drugs – may improve the clinical outcome of psychiatric patients.

## HALLMARK 6: HOMEOSTATIC RESILIENCE

**Figure 6:**
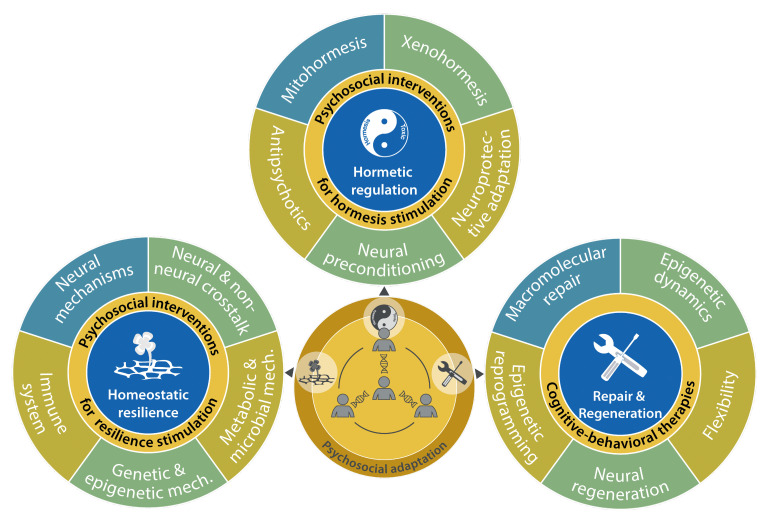
FIGURE 6: Psychosocial dimension of the hallmarks of health involved in responses to stress. Interactions between psychosocial adaptation and mechanisms of homeostatic resilience, hormetic regulation and repair and regeneration strategies aimed at achieving biological stability and maintenance of health, including mental health.

Homeostatic regulation defines resilience and determines lifespan and healthspan by controlling and repairing internal damages, eliciting appropriate stress responses, minimizing biological noise, and facilitating constant tissue remodeling [1]. Homeostatic resilience involves the participation of complex neural mechanisms, which act in concert with a variety of genetic, epigenetic, metabolic, endocrine, immunological and microbial processes. Deficiencies in any of these resilience mechanisms may contribute to the development and progression of numerous human pathologies, including mental disorders **(Fig. 6)**.

### Neural Mechanisms

Resilience results from adaptive changes in the functional activity of numerous brain circuits that control the psychobiological responses to acute or chronic stressors. These changes involve the participation of multiple hormones, neuropeptides, neurotransmitters, and their corresponding receptors and signaling pathways, which collectively elicit homeostatic responses to stress [196]. Acute actions of glucocorticoids are protective and elicit homeostatic resilience responses, whereas chronic exposure to high glucocorticoid levels causes neural damage and debilitates mental health [197]. Glucocorticoids function in close coordination with neurotransmitters and neurotrophic peptides to modulate stress resistance. Acute stressors increase the brain turnover of serotonin, a central component of the circuits that control mood and emotion. They also affect dopamine, which modulates reward and aversion, contributes to fear extinction and participates to stress resilience [198]. Neuropeptide Y (NPY) is another factor that induces anxiolytic effects under stressful conditions [199]. In patients with MDD or PTDS, plasma levels of NPY are reduced [200], again illustrating the diversity of neural mechanisms of homeostatic resilience.

### Neural-Non-Neural Crosstalk

Homeostatic resilience is not only directly controlled by neuron-intrinsic mechanisms, but also involves non-neuronal cells (i.e., glial, myeloid and endothelial cells) that interact within the CNS to modulate neural networks and control stress behaviors. Non-neuronal cells from limbic regions of brain can interact at synapses, the neurovascular unit, and other sites of cell-cell communication to mediate both the pro-resilient and deleterious effects of chronic stress [201]. These brain regions play critical roles in the regulation of mood and emotional states through a fertile crosstalk between neural and non-neural components. For example, in response to aversive or rewarding stimuli, the nucleus accumbens integrates dopaminergic projections from the ventral tegmental area and glutamatergic inputs from the hippocampus, prefrontal cortex, amygdala and thalamus, and then determines resilience to stress, as well as reward-driven learning and motivation [202]. Similarly, a stress-sensitive projection connecting basolateral amygdala and nucleus accumbens plays a critical role in executing disrupted reward behaviors provoked by early-life adversity [203]. The correct function of the hippocampus-amygdala complex and its interaction with the prefrontal cortex are also essential for resilience mechanisms aimed at the control of intrusive memories caused by trauma [204]. Likewise, stress and glucocorticoid release decrease adult neurogenesis at the dentate gyrus, and increasing neurogenesis in mice indeed promotes resilience to social defeat stress by inhibiting the activity of mature granule cells [205]. A specific circuit in the midbrain involving GABA-somatostatin producing cells detects stress and induces restorative sleep [206], while overexpression of the zinc finger protein gene *Zfp189* in the prefrontal cortex promotes behavioral resilience [207]. Also in this regard, Dong *et al.* have recently identified stress relief as a natural resilience mechanism against depression-like behaviors [208].

Alterations in these communication systems contribute to stress-related disorders by compromising a broad range of processes such as adequate differentiation and maturation of oligodendrocytes and astrocytes, limited trafficking of peripheral myeloid cells to the brain, maintenance of ionic and neurotransmitter homeostasis, proper dynamics at synapses and preservation of BBB integrity. Stress-related signals such as oxidative reactions and cytokine signaling from the periphery may induce the loss of myelin in brain areas related to emotional regulation and executive function. Neurovascular adaptations, endocannabinoid signaling and the kynurenine pathway also contribute to modulate mood and stress responses [209–211].

### Immune System

Psychosocial stress strongly influences immune function and modulates the participation of immune cells in homeostatic resilience mechanisms. Different brain regions and neural circuits control the body trafficking and functional role of leukocytes in response to stress [212]. Motor circuits trigger the rapid mobilization of bone marrow neutrophils to peripheral tissues through the participation of neutrophil-attracting chemokines, while the hypothalamus controls the egression of lymphocytes and monocytes from blood and secondary lymphoid organs to the bone marrow via glucocorticoid signaling. These stress-induced changes in leukocyte distribution throughout the body are linked to the development of several disorders including mental illnesses. Some patients with stress-related disorders exhibit increased peripheral immune system activation and elevated levels of proinflammatory cytokines, which then activate the kynurenine pathway, depleting tryptophan and generating neuroactive catabolites that impinge on the main stress response pathways [211].

Chronic stress can also directly activate microglia and increase levels of several cytokines and chemokines through glucocorticoid and noradrenergic signaling or via the NLRP3 inflammasome. Notably, MDD exhibits significant comorbidity with autoimmune disorders and other chronic inflammatory illnesses. Accordingly, anti-inflammatory therapies elicit antidepressant effects in some patients [37]. Animal models of social stress have also shown important disturbances in peripheral myeloid cells, which are associated with activation of the innate immune system and relative suppression of the adaptive immune system [213]. Rodent studies of susceptibility to chronic stress are consistent with a pro-resilient neuroprotective effect of T cells. Immunization of rats with modified myelin basic protein before chronic mild stress induces autoreactive T cells and reduces depressive behaviors [214]. Resilience to stress can also be promoted by immunization against *Mycobacterium* [215], or by attenuation of inflammation via sphingosine-1-phosphate receptor 3 (*S1PR3*) overexpression in the medial prefrontal cortex of rats [216]. Finally, clinical studies have confirmed that emotion regulation strategies can attenuate inflammatory responses [217].

### Genetic Mechanisms

Susceptibility or resilience to develop behavioral disorders in response to psychosocial stress is influenced by the interplay between genetic predisposition and environmental factors. Genomic investigations identified pro-resilience variants in genes encoding modulators of the HPA stress response axis. Thus, a polymorphism in *FKBP51* – a negative modulator of glucocorticoid signalling – is linked to susceptibility (AT allele) or resilience (CG allele) to stress-related disorders. Pharmacological inhibition of FKBP51 promotes hippocampal neurogenesis and resilience to chronic psychosocial stress in mice [218]. Additionally, polymorphisms in *NPY, BDNF, COMT* and *SLC6A4* associated with deficient resilience increase the risk of mental illness [219]. Notably, the same genomic variants (i.e., polymorphisms at the regulatory region of the serotonin transporter gene *SLC6A4*) that increase the risk of pathological responses to adversity may be beneficial in favorable environments [220]. This “pleiotropic antagonism” reflects the fact that, depending on the context, the same genetic variant can have positive or negative consequences.

### Other Resilience Mechanisms

The initial findings linking epigenetic alterations and MDD were based on the observation that loss or inhibition of histone deacetylases and demethylases in several brain regions has antidepressant-like effects in stressed rodents [221]. Moreover, glucocorticoids suppress DNA methylation and upregulate the expression of the stress-response gene *Fkbp5* in mouse neuronal cells, thus generating a negative feedback loop that limits glucocorticoid signaling and may contribute to stress-related mental disorders [222]. Several miRNAs, such as miR-25-3p, are induced in mice exposed to social defeat stress. Selective elimination in peripheral leukocytes of the miRNA cluster containing miR-25-3p reduces inflammation and promotes behavioral resilience to psychosocial stress [223]. miR-135 is necessary for maintaining intact serotonergic activity under normal conditions and confers resilience to social stress [224], whereas overexpression of miR-124 in hippocampal neurons enhances chronic stress resilience [225]. Likewise, systemic knockdown of *miR-144-3p* by subcutaneous administration of a specific antagomir reduces the depression-related phenotype in stress-susceptible mice [226]. Together, these works reinforce the role of epigenetic mechanisms in inflammatory and behavioral responses to psychosocial stress.

Hormonal and metabolic pathways also influence stress resilience, and patients with stress-induced psychiatric disorders exhibit metabolic phenotypes that substantially overlap with metabolic syndrome [227]. Glucocorticoids switch metabolism from anabolism to catabolism, thereby providing energy sources and building blocks for adequate stress responses. These glucocorticoid effects are modulated by other hormones, such as leptin and ghrelin [228, 229]. Somatostatin also contributes to resilience by reducing CRH release during chronic stress conditions. Sex hormones have a strong impact on homeostatic resilience and explain, at least in part, the sexual dimorphism in the responsiveness to chronic stressors [230]. Finally, microbiota-related mechanisms also contribute to stress resilience. The maintenance of a stable microbiota contributes protects from a variety of dysbiosis-related pathologies but is also critical for establishing the cognitive/emotional balance necessary to deal with psychosocial stress. In fact, alterations in the gut microbiota have been detected in a variety of mental disorders, such as MDD and ASD. The healthy microbiota contributes to homeostatic resilience against stress conditions through the production of biologically active metabolites impacting the microbiota-gut-brain axis [231]. Diverse prebiotics, probiotics and postbiotics may increase the resilience of gut bacterial communities, although for most of the currently available products there is no clear evidence yet to support beneficial effects on mental health [232].

In summary, highly interconnected body communication systems are organized in a way that allows them to elaborate a rapid and efficient response to virtually any kind of perturbation. These responses mostly involve negative feedback loops and are ultimately responsible for maintaining homeostatic resilience. Unfortunately, a wide range of chronic or excessive stressors cause the failure of resilience mechanisms and promote neuropsychiatric decompensation. Further studies of the mechanisms underlying homeostatic resilience and failure should help to design interventions that favor the maintenance of mental health.

## HALLMARK 7: HORMETIC REGULATION

Hormesis is an evolutionary conserved phenomenon that leads to the development of acquired resilience against toxins and other stressors called hormetins [233]. Hormesis relies on biological processes in which low doses of potentially harmful agents elicit a protective response that prevents the organism from experiencing harm upon exposure to a higher dose of the same hormetins. Hormesis has been pinpointed in the context of mitochondrial function as “mitohormesis” to describe the beneficial effects of mild and transient mitochondrial stress on cells, tissues or organisms [234]. Mitohormesis inducers include physical exercise, caloric restriction, intermittent fasting, and dietary phytochemicals or xenohormetins [235]. The beneficial effect of hormesis may rely on direct short-range cytoprotection through the induction of ROS, heat shock proteins, thioredoxins and sirtuins, but may also involve long-range intercellular communication events via neural circuits, endocrine signals, metabolic pathways, and immune or inflammatory responses [233]. Hormesis is also involved in the maintenance of mental and brain health **(Fig. 6)**.

### Hormesis and Psychotropic Drugs

The concept of hormesis may offer a useful framework to improve neurological performance and brain health [236]. Embryonic, adult and induced-pluripotent stem cells of different sources, including those of neural origin, exhibit hormetic responses to low doses of noxious chemicals, ionizing radiation and hypoxia with respect to their capacities to proliferate, differentiate and resist inflammatory conditions [237, 238]. Dietary supplements reputed to improve human health, such as epigallocatechin-3-gallate and resveratrol, may also induce hormetic responses in neural stem cells [238], while lithium, a widely used drug for the treatment of BD and other mood-related disorders, elicits biphasic dose responses typical of hormesis [239].

Some neurotoxic agents induce reactive oxygen species (ROS) that at low levels activate hormetic responses in stressed neurons and induce the expression of genes – such as *BCL2* and *SOD2* – which protect against apoptosis and detoxify ROS, respectively. Downstream of ROS, transcription factors – such as NRF2 – trigger efficient cytoprotective mechanisms [240]. The endogenous metabolite N-acetyl-L-tyrosine (formed in response to stress from its precursor tyrosine) triggers a mitohormetic process that implies an elevation of ROS levels and a subsequent retrograde response activating the transcription factor FoxO, which in turn transactivates anti-oxidant genes and KEAP1 to elicit neuroprotective mechanisms [241]. Atypical antipsychotic drugs may also act through hormetic mechanisms and mediate neuroprotection through the induction of superoxide dismutase 1 and p75 neurotrophin receptor [242, 243]. Peripheral modulation of the antidepressant targets MAO-B and GABA_A_R by β-carbolines induces mitohormesis and improves healthspan and lifespan in preclinical models [244]. These findings suggest that several classes of antipsychotic drugs elicit hormetic effects, although it is unclear whether this truly contributes to their mode of action.

### Hormesis and Mental Stress

Mild and limited stress can result in a series of moderate cognitive benefits that facilitate the development of human resilience [245]. Accordingly, hormesis has been proposed to play a positive role in cognitive processes and behavioral responses [246]. In favor of this interpretation, cortisol concentrations measured in adolescents were the lowest in individuals experiencing moderate socioeconomic and psychosocial adversity, but higher in individuals reporting low or high adversity [247]. Moreover, low-to-moderate stress perceived by young adults correlates with optimal cognitive performance and reduced psychopathological symptoms [248]. Thus, in a well-tempered/medium range, negative life experiences and perceived stress may have a beneficial effect.

From an educational/psychological viewpoint, it appears important to change the valuation of stress by shifting the overarching objective of stress regulation from avoiding and minimizing stress to accepting and utilizing stress to achieve enhancing outcomes [31]. The subjective appraisal of stress as negative (distress) versus positive (eustress) has a profound impact on its consequences, which can be detrimental versus hormetic, respectively. Indeed, negative beliefs about stress constitute an independent risk factor for morbidity and mortality [249], in line with the well-established negative impact of pessimism on life expectancy [250]. Several randomized studies with adolescent have proven that psychological training designed to improve the acceptance of stress reduced cortisol levels and perceived anxiety [31]. Notably, improved stress management correlates with higher emotional intelligence [251], while optimal stress responses may explain “post-traumatic growth”, a phenomenon allowing individuals to develop increased skills and a deeper appreciation for life as a legacy of traumatic events [252].

In summary, the concept of hormesis has gained interest in the field of neural functions. The use of hormetic preconditioning strategies can enhance the functional performance of neural cells, including neural stem cells, with respect to their ability to improve metabolic functions and contribute to neuroplasticity, neurorepair or regeneration. Theoretically, knowledge on hormetic regulation may help to establish optimal schedules for administering drugs that favor brain health and cognitive performance.

## HALLMARK 8: REPAIR AND REGENERATION

Organisms have developed complex mechanisms and signaling pathways able to sense and efficiently respond to the myriad of lesions suffered by all organizational strata of the body, from molecules to the meta-organism, and to activate repair and regeneration mechanisms [172]. Insufficient repair and regeneration entails a broad range of pathological perturbations, including those causing neuropsychiatric disorders **(Fig. 6)**.

### DNA Damage and Repair in Neural Systems

Nuclear and mitochondrial DNA are constantly subjected to genotoxic stress by exogenous and endogenous challenges. This causes a wide spectrum of DNA lesions, which are repaired by a network of systems collectively known as the DNA damage response (DDR) [253]. The effectors of this response, such as TP53 and various immune cells, drive cellular senescence or apoptosis and contribute to maintain homeostasis. DDR also engages the cGAS/STING pathway and stimulates a non-cell-autonomous response that facilitates homeostasis maintenance. However, deregulated DDR causes uncontrolled inflammation and tissue damage, including in the CNS [254, 255]. Impaired DNA damage repair in concert with mitochondrial dysfunction is a common feature of diverse psychiatric disorders [255, 256]. Elevated levels of oxidative DNA damage and altered DNA repair gene expression are found in GABAergic neurons in SCZ, while genomes from ASD patients are enriched for de novo mutations in genes expressed in striatal neurons [257]. Interestingly, selective serotonin reuptake inhibitors decrease the level of oxidative DNA damage in MDD patients [258]. Hence, it will be interesting to test pharmacological agents that stimulate oxidative DNA damage repair [259] in the context of neuropsychiatric disorders.

### Epigenetic Dynamics, Reprogramming and Mental Health

Epigenetic factors contribute to the development and promotion of neurological and behavioral diseases [260]. The expression of the epigenetic reader BRD1 increases after periods of chronic stress, and *Brd1*^*+/-*^ mice display cognitive deficits and behavioral phenotypes [261]. Chromatin profiling in neurons from SCZ patients has revealed aberrant roles for histone acetylation and BRD1 [262], while BRD1-interaction networks show enrichment for SCZ risk genes and enhanced binding to gene promoters associated with brain development and mental disorders [261]. Besides histone modifications, DNA methylation studies in postmortem SCZ brains have identified multiple differentially methylated sites between cases and controls. Genes in or near these sites tend to be involved in embryo development, cell fate commitment or nervous system differentiation, and are also modestly overrepresented in SCZ-associated loci. MDD patients exhibit higher global DNA methylation rates than healthy controls and a significant correlation of gene methylation changes in the blood and in MDD-relevant brain areas such as the prefrontal cortex [263]. DNA methylation-based epigenetic clocks that reflect biological aging indicate that MDD patients undergo accelerated aging compared to non-depressed controls [264], while individuals with different behavioral disorders exhibit an accelerated pace of DNA-methylation [265]. Additionally, correlative studies have found significant ncRNA alterations during stress-induced responses and in patients with mood disorders. Knockout of the SCZ-related *miR-501-3p* gene in mice impairs sociability and memory by enhancing mGluR5-mediated glutamatergic transmission, while treatment of these *mir-501-3p-*null mice with negative allosteric modulators of mGluR5 or NMDA receptor antagonists ameliorates their cognitive and behavioral deficiencies [266].

Collectively, these findings suggest that neuropsychiatric disorders are linked to epigenetic alterations, opening new therapeutic strategies aiming at restoring the epigenetic landscape [267]. Indeed, epigenomic editing at the enhancer region of the activity-regulated cytoskeleton-associated protein (*Arc)* gene in rats ameliorates adult anxiety and excessive drinking after adolescent alcohol exposure, a major risk factor for psychiatric disorders later in life. Conversely, dCas9-KRAB increases repressive histone methylation at this genomic region, decreases *Arc* expression, produces anxiety and stimulates alcohol drinking in control rats [268].

### Neural Regeneration and Mental Health

Stem and progenitor cells can repair or regenerate damaged tissues and hence favor adaptive and compensatory responses. Stem cells are also present in the mammalian brain, an organ long-time considered to be irreparable. Adult neurogenesis has been well characterized in the dentate gyrus of the rodent hippocampus and has important implications for regenerative medicine in humans, although the possibility that this process is fully preserved in the adult human brain is still debated [269–272]. Neural stem cells can self-renew and generate terminally differentiated neurons and glial cells. Due to their persistence throughout life, stem cells are particularly susceptible to biological and environmental stress, and decline in number and proliferative and differentiation capacity with age, compromising tissue repair and regenerative potential [108, 109]. Several studies have suggested that psychosocial factors may contribute to stem cell loss [273]. Moreover, chemogenetic inhibition of neurogenesis in the ventral dentate gyrus promotes susceptibility to social defeat stress, while increasing neurogenesis confers resilience to chronic stress [205]. Notably, treatment with atypical antipsychotics increases hippocampal neurogenesis in adult mice [274]. In MDD patients, neurotrophic factors necessary for neural stem cells niche maintenance are reduced, while low levels of neurotrophic factors have been associated with poor treatment responses and cognitive impairment in MDD [275].

Physical exercise and dietary interventions may also contribute to neurological repair. Several signaling circuits including glutamatergic, serotonergic, dopaminergic, adrenergic, neurotrophin-receptor and tropomyosin-related kinase B pathways have been implicated in the exercise-stimulated enhancement of neurogenesis [276]. In addition, regulator of G protein signaling 6 stands out as a key mediator of exercise-induced neurogenesis [277]. The administration of exerkines (molecules released in response to physical exercise) [278]. and exercise mimetics (compounds that mimic the therapeutic effects of exercise) [279]. may represent emerging strategies for improving neurogenesis and synaptic plasticity [280]. Intermittent fasting enhances long-term memory consolidation, adult hippocampal neurogenesis, and expression of the longevity gene *Klotho* [281]. Consistent with this, low-dose injections of Klotho increase synaptic plasticity in mice and improve cognition in aged nonhuman primates [282]. Similarly, caloric restriction and diets enriched with bioactive compounds, such as polyunsaturated fatty acids and polyphenols, improve neurogenesis, learning and memory performance in neuropsychiatric diseases [283].

### Cognitive and Behavioral Flexibility

Flexibility is substantially impaired across many mental disorders irrespective of the age of onset [284]. Neuroimaging, behavioral, genetic and pharmacological studies [285] have identified large functional brain networks that support flexibility. Serotonergic and dopaminergic signaling, as well as striatal cholinergic systems play important roles in flexible cognition and behavior. Reduced neurogenesis, changes in dendritic morphology and density, and alterations in growth factor and neurotransmitter levels contribute to the loss of neuroplasticity and functional connectivity underlying cognitive and behavioral inflexibility in mood-related disorders [285]. Social isolation reinforces aging-related behavioral inflexibility in aging-prone SAMP8 mice by promoting neuronal necroptosis (an alternative mode of regulated cell death mimicking features of apoptosis and necrosis) in basolateral amygdala, a critical brain region for behavioral flexibility [286]. This flexibility impairment can be reversed by the necroptosis inhibitor necrostatin-1s, and involves inhibition of glycogen synthase kinase 3*α* (*GSK-3α*), a central regulator of age-related pathologies in mice [286]. Interestingly, infusion of young cerebrospinal fluid into brains of aged mice restores oligodendrogenesis and memory through a process involving Fgf17, thereby offering new possibilities to enhance cognitive flexibility [287]. Of note, and in the context of tissue damage, preemptive immunity to the microbiota directly promotes neuron regeneration via IL-17A [288]. Other strategies to improve behavioral flexibility include antidepressant drugs, lifestyle interventions, enrichment of the particular social environment and different methods of cognitive training [289, 290].

## CONCLUSIONS AND PERSPECTIVES

**Figure 7:**
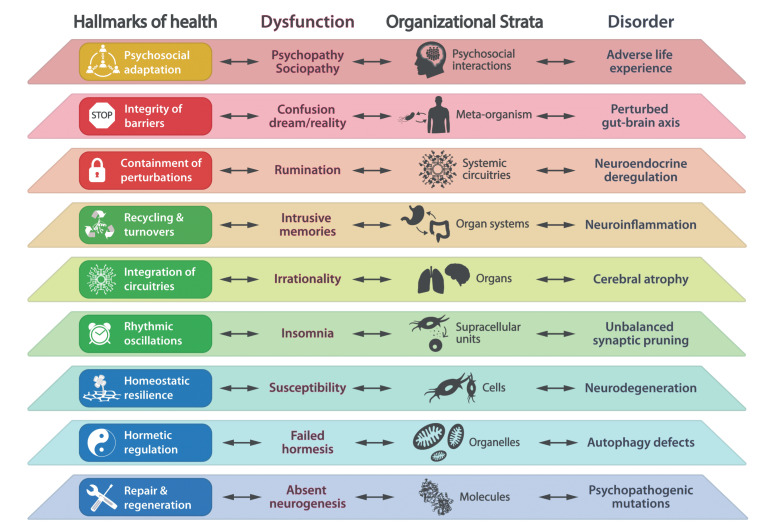
FIGURE 7: Psychosocially relevant perturbations across health hallmarks and body strata. The nine hallmarks of health integrate and leverage the multifunctionality of each hierarchical stratum and orchestrate the complex interactions across the nine body strata encompassing the distinct molecular, organellar, cellular, supracellular, tissular, systemic, organismal, meta-organismal and psychosocial components. Examples of specific psychosocial dysfunctions or disorders affecting the nine different hallmarks and strata are shown to illustrate the multidimensional basis of health, including mental health.

To advance in the comprehension of human health, which does include essential mental facets, we have expanded our previous compendium of eight biological hallmarks [1] by adding a ninth determinant of health, psychosocial adaptation. In addition, we have analyzed the psychosocial, mostly neuropsychiatric, implication of each of the eight somatic hallmarks, unveiling numerous connections between all somatic hallmarks and psychosocial factors. We have also identified links between mental health and all eight somatic strata of the human body, from individual molecules through cells and tissues to the meta-organism, in addition to the stratum encompassing psychosocial interactions. Perturbations of each of these nine strata threaten mental health **(Fig. 7)**.

The inclusion of the psychosocial dimension as a further hallmark of health is in accordance with earlier models of health and disease [291], and with current definitions and concepts of health and well-being [292]. Nevertheless, our work has tried to go beyond these studies by integrating psychosocial and biological determinants of health. We have shown that there are so many intricate links between the somatic and psychosocial hallmarks of health that the contours of physical and mental health dissipate. We posit that virtually any disease, even pathologies with an infectious or mechanical cause have a major effect on the mental state, while all major mental disorders compromise general health. This holistic view transcends the unidirectional idea of “mens sana in corpore sano”, which correctly expresses the notion that physical fitness is required for mental agility but does not reflect the fact that, vice versa, psychosocial adaptation is indispensable for somatic health.

The historical distinction between physical and mental diseases has been fueled by the apparent absence of a clear anatomopathological substrate of the latter. However, progress in ever more refined “omics” technologies yielding a “brain atlas” [293], the incipient elucidation of the neural connectome [294], and the development of high-resolution functional neuroimaging tools, has yielded quantitative measurements of CNS perturbations. Ultimately, these objective measurements will create a mechanism-based classification of neuropsychiatric disorders and will drive the era of precision and preventive psychiatry [295], which will have to be integrated with cultural-ecosocial approaches aimed at person-centered mental health care [296]. The progress in analytical tools will be coupled to powerful experimental systems including multiplexed chemogenetic or optogenetic methods, and to the development of sophisticated iPSC-derived human cerebral organoids that are able to maturate and integrate with host circuits controlling behavior when implanted into rodent brains [297]. Such experimental approaches will also be indispensable for structuring AI-based algorithms and hence for interpreting the accumulating big data generated by large-scale “omic” efforts as well as by the monitoring of human behavior, either by observation or by means of specific tests. Systematic approaches will also be necessary to quantify the exposome that, besides the totality of physical, chemical, dietary, microbial and toxicological insults, should also integrate psychological and social stressors. Of note, there is evidence of association between exposure to environmental pollutants and the incidence of dementia and behavioral disorders, again linking somatic to psychosocial factors [24]. This question is of special concern in the context of the current climate change that is going to impose grand challenges for future human health, including mental health [298]. We also need to better understanding how psychological, social and economic stress permeates the human body. Beyond the frequently evoked SAM and HPA axes, additional communication systems linking the CNS to the periphery are likely to play a major role in this process. The elucidation of these pathways might guide neuropsychiatric research from the current CNS-centric to a body-wide exploration.

Today, around one billion people are affected by mental disorders [299] and dramatically, close to one million individuals decide to take their own lives each year [300]. However, the relative investment in mental health services is under-dimensioned compared to physical health systems [24]. Over the last decades, the extraordinary progress of science and medicine has resulted in remarkable advances in the prevention or treatment of most human pathologies, but progress has been more limited with regard to psychosocial health [155]. Several studies have provided evidence that certain psychosocial interventions improve physical health [27–30], but it is necessary to perform large-scale, long-term randomized clinical trials to assess the specific benefits provided by these interventions. Similarly, public campaigns to promote population health must expand their current focus on exercise, diet, environmental pollution, alcohol and drug abuse, and target additional lifestyle factors that favor psychosomatic health. Such neglected lifestyle factors are exemplified by the need of increased levels of social respect and education, the avoidance of digital addiction, the improvement of quantity and quality of sleep, the introduction of mind-body and stress management practices, and – utopically but necessarily – the reduction of violence and inequality.

## Abbreviations:

WHO: – world health organization,
ASD: – autistic spectrum disorders,
MDD: – major depressive disorder,
BD: – bipolar disorder,
SCZ: – schizophrenia,
SAM: – sympathetic-adrenal medullary,
HPA: – hypothalamic pituitary adrenal,
IL6: – interleukin-6,
Tac2: – tachykinin 2,
mtDNA: – mitochondrial DNA,
iPSC: – induced pluripotent stem cell,
BBB: – blood-brain barrier,
CNS: – central nervous system,
CLDN5: – claudin-5,
HDAC1: – histone deacetylase 1,
FOXO1: – forkhead box protein O1,
C4A: – component 4A,
EBP1: – ErbB3-binding protein 1,
FMRP1: – fragile X mental retardation protein 1,
DISC1: – disrupted-in-SCZ-1,
CSF1R: – colony stimulating factor 1 receptor,
NLGN3: – neuroligin-3,
NPY: – neuropeptide Y,
S1PR3: – sphingosine-1-phosphate receptor 3,
ROS: – reactive oxygen species,
DDR: – DNA damage response,
Arc: – activity-regulated cytoskeleton-associated protein,
GSK-3α: – glycogen synthase kinase 3α.
